# Predicting Depression Severity in Major Depressive Disorder Using Electroencephalography and Heart Rate Variability: A Cross-Sectional Analytical Study

**DOI:** 10.7759/cureus.79798

**Published:** 2025-02-27

**Authors:** Praveen Prakash, Prabhu Natesan, Rajalakshmi Rajasegaran, G. K. Pal, Vikas Menon

**Affiliations:** 1 Department of Physiology, Jawaharlal Institute of Postgraduate Medical Education & Research, Puducherry, IND; 2 Department of Psychiatry, Jawaharlal Institute of Postgraduate Medical Education & Research, Puducherry, IND

**Keywords:** electroencephalography (eeg), frontal alpha asymmetry, heart rate variability (hrv), major depressive disorder (mdd), psychiatric biomarkers

## Abstract

Introduction

Mood disorders comprise the largest burden on mental health, with major depressive disorder (MDD) being the most prevalent among them. While the diagnosis of mood disorders is generally subjective based on history and structured interviews or questionnaires, efforts are being made to identify biological markers that could provide an objective method for diagnosis, classification, prognosis, and elucidation of the underlying mechanisms of mood disorders. Electroencephalography (EEG) and heart rate variability (HRV) are two biosignals that have been studied in this regard. This study aimed to assess the associations between the severity of MDD, EEG alpha asymmetry, and HRV indices in individuals diagnosed with MDD. The EEG and HRV findings were also compared with age- and gender-matched euthymic controls.

Methods

In this cross-sectional study, 45 individuals (28 males and 17 females, aged 18-50 years) with MDD (as per the Diagnostic and Statistical Manual of Mental Disorders, Fifth Edition) who were either drug naive or had a drug-free interval of at least six weeks (defaulters) were included in the test group. Similarly, 45 age- and gender-matched healthy individuals were included in the control group. The study parameters, namely, the severity of depression, EEG alpha asymmetry, and HRV indices, were assessed from the Hamilton Depression Rating Scale 17 (HDRS-17) score, EEG recording, and lead II ECG data, respectively, and were compared across the test and control groups. In addition, the bivariate correlation between the severity of depression and other parameters in the MDD group and linear regression modeling with the severity of depression as the dependent variable and EEG alpha asymmetry and HRV indices as predictors were performed for the test group.

Results

HRV indices, namely LF n.u. and LF/HF ratio, were significantly higher, and HF n.u. was significantly lower in MDD individuals compared to healthy controls. A comparison of alpha asymmetry metrics revealed significantly high alpha asymmetry in MDD individuals compared to controls in all three frontal lead pairs (Fp1-Fp2, F3-F4, and F7-F8) and the T3-T4 temporal lead pair. While weak positive correlations were observed between HDRS-17 score and age (ρ = 0.313, p = 0.036), illness duration (ρ = 0.336, p = 0.024), F7-F8 asymmetry (r = 0.364, p = 0.014), LF n.u. (r = 0.349, p = 0.019), and LF/HF ratio (ρ = 0.416, p = 0.005), a weak negative correlation was observed between HDRS-17 and HF n.u. (r = -0.347, p = 0.019) in the test group. Similarly, age, illness duration, F7-F8 asymmetry, O1-O2 asymmetry, LF n.u., HF n.u., and LF/HF ratio were identified as correlates of HDRS-17 for multiple linear regression modeling. The resulting model, with F7-F8 asymmetry and HF n.u. as predictors, was found to be statistically significant in the test group (R² = 0.224, p = 0.005).

Conclusions

Evidence of a possible relationship between the severity of depression and two objective physiological measures, namely HRV HF n.u. and EEG frontal alpha asymmetry (FAA), was observed in this study. The combination of HRV HF n.u. and EEG F7-F8 FAA could explain 22.4% of the variation in severity of depression, as quantified by HDRS-17, mainly in newly diagnosed drug-naive patients of MDD at presentation.

## Introduction

Major depressive disorder (MDD) is a psychiatric disorder characterized by persistent low mood, anhedonia, and other associated symptoms that cause significant distress and impairment in daily life. The global prevalence of MDD stood at an estimated 229 million in 2021, of which 45.4 million were in India [1]. Mood disorders are generally diagnosed in a subjective manner using structured interviews and eliciting psychological and behavioral history [2]. Due to the inherent subjectivity, mood disorders are challenging to diagnose. Complicating this is the lack of consensus surrounding the classification, diagnosis, and treatment that is rooted in the limited and incomplete understanding of the underlying psychopathophysiology [2]. Hence, there is an ongoing attempt to identify biological markers that could provide an objective basis for the diagnosis and classification of mood disorders as well as further elucidate the underlying mechanisms of their pathophysiology [2]. Electroencephalography (EEG), a noninvasive technique for recording electrical activity of the brain via scalp electrodes, has gained attention in this regard as a minimal risk procedure with high temporal resolution, enabling precise evaluation of both baseline and evoked responses [3].

The literature describes several potential markers, among which alpha asymmetry has been studied commonly. Alpha asymmetry in the frontal and parietal EEG leads has been reported as a potential discriminator between depressed and euthymic individuals [4,5]. However, studies in the recent past have reported inconclusive and contradictory findings regarding alpha asymmetry and other EEG markers in depression [3,4]. This has been attributed to the heterogeneous nature of patients with MDD as well as a variety of postulated confounding factors [3].

In addition to EEG biomarkers, studies show heart rate variability (HRV) measures to be significantly lower in subjects with major depression [6]. HRV refers to the physiological variation in time intervals between consecutive heartbeats, typically measured as the R-R intervals on an electrocardiogram (ECG). It reflects cardiac autonomic function, particularly the balance between sympathetic and parasympathetic activity [6]. Evidence suggests that HRV might serve as an indicator of the effectiveness of the prefrontal cortex in managing emotional responses, adaptability to various psychological states, and social engagement, all three of which show impairments in mood disorders like MDD [7,8]. Both HRV and EEG biomarkers have shown limited utility as diagnostic markers [3,6]. Setting aside diagnosis, we wished to ascertain if the combination of a neurophysiological measure (EEG) with a measure of autonomic function (HRV) could predict the severity of depression in already diagnosed patients, particularly in the drug-naive or drug-free state. Such associations can contribute to the development of more objective prognostic and classification tools. Hence, the primary objective of this work was to explore the associations between the severity of depression, EEG alpha asymmetry, and HRV indices in MDD patients who are not on psychiatric medication at the time of measurement. As a secondary objective, we recruited age- and gender-matched controls to compare our EEG and HRV findings with euthymic individuals.

## Materials and methods

This cross-sectional analytical study was conducted from July 2021 to July 2023 in the Departments of Physiology and Psychiatry at the Jawaharlal Institute of Postgraduate Medical Education & Research in Puducherry, India, following approval from the Postgraduate Research Monitoring Committee and the Institute Ethics Committee (JIP/IEC/2021/182).

The study sought to recruit patients with MDD, as defined by the Diagnostic and Statistical Manual of Mental Disorders, Fifth Edition (DSM-5) [9], in the age group of 18 to 50 years, who were drug naive or had a drug-free interval of at least six weeks at the time of recording. The DSM-5 defines MDD as a cluster of symptoms that occur for at least two weeks and represent a change from the individual's previous functioning. The core symptoms of MDD include “a depressed mood or loss of interest or pleasure in most activities, as well as a range of other cognitive, emotional, and physical symptoms such as feelings of worthlessness or guilt, changes in appetite or weight, sleep disturbances, fatigue or loss of energy, difficulty concentrating or making decisions, and recurrent thoughts of death or suicidal ideation.” To meet the criteria for MDD, an individual must experience at least five of these symptoms, including either a depressed mood or loss of interest or pleasure, and the symptoms must cause significant distress or impairment in social, occupational, or other areas of functioning. Additionally, the symptoms should not be better accounted for by another medical condition or substance use [9].

Through convenience sampling, approximately 200 patients diagnosed with MDD were selected from the psychiatry outpatient department and subjected to a screening interview. A total of 45 cases were found eligible and included in the study. Among these, 43 patients were newly diagnosed and self-reported to be drug naive at the time of recording, while two were established cases who had defaulted on their medication (both individuals had been prescribed fluoxetine and clonazepam) and had a self-reported drug-free interval of at least six weeks, which was corroborated against the records of their previous visit. However, individuals with comorbid medical, surgical, neurological, or other psychiatric illnesses and those on long-term medications that could alter autonomic functions were excluded from the study.

For controls, 45 healthy age- and gender-matched individuals were recruited from the local community through convenience sampling using referrals and outreach after screening for symptoms of MDD and other major psychiatric illnesses through interview. Written informed consent was obtained from all participants prior to recruitment.

Assessment of depression

The severity of depression of each individual was assessed using the 17-term clinician-rated Hamilton Depression Rating Scale (HDRS-17) using a semi-structured interview [10]. All participants were interviewed by the same rater who was trained in applying the scale. Scores range from 0 to 52, with scores 0-7 indicating no depression, 8-16 mild depression, 17-23 moderate depression, and over 24 severe depression.

EEG and ECG acquisition

EEG and EEG were recorded simultaneously in the neurophysiology laboratory following standard guidelines. Recording was done between 10 AM and 12 PM to control for general circadian variation in measured signals. All participants were asked to refrain from caffeine and nicotine use for 24 hours prior to the recording. The lab was air-conditioned to maintain a room temperature of 24°C and ensured a private and quiet environment. Participants were made to lie comfortably in a semi-recumbent position and were instructed to minimize eye and body movement. An appropriately sized electrode cap was placed using the international 10-20 system of electrode placement as recommended by the International Federation of Societies of EEG and Clinical Neurophysiology. Electrode input impedance was minimized to less than 5 kΩ. EEG signals were recorded through 21 Ag/AgCl electrodes using the Galileo Systems B.E. Light EEG platform (EB Neuro S.p.A., Firenze, Italy). After the preparation, participants were given five minutes of rest, after which resting baseline EEG was recorded in the eyes-closed state for a total recording duration of 10 minutes. Simultaneously, a lead II ECG was also recorded using a bipolar amplifier channel built into the EEG platform, which was extracted and used to derive short-term HRV parameters. The subjects were closely monitored to ensure that they were alert during the recording.

Analysis of EEG alpha asymmetry

Raw EEG signals were acquired at a sampling frequency of 512 Hz. EEG filtering, artifact rejection, and cleaning were performed with Brainstorm version 3.211012 [11], an open-source EEG analysis software under the GNU general public license. Preprocessing involved the following sequential operations: application of a high-pass filter with a threshold frequency of 1 Hz, application of a notch filter centered at 50 Hz, application of a low-pass filter with a threshold frequency of 100 Hz, events/labels created at two-second intervals (zero seconds, two seconds, four seconds, six seconds, etc.), and dividing the entire EEG recording into two-second segments.

From the preprocessed data, segments with artifacts were removed by visual inspection, and the artifact-free segments were exported. EEG frequency bands were defined as follows: Delta (δ) Band - 2 to 4 Hz; Theta Band (θ) - 5 to 7 Hz; Alpha Band (α) - 8 to 12 Hz; Beta Band (β) - 15 to 29 Hz; Gamma1 Band (γ1) - 30 to 49 Hz; Gamma 2 Band (γ2) - 51 to 98 Hz. For this study, data pertaining to the alpha band was analyzed further. Cleaned and preprocessed data files were analyzed using MNE-Python version 1.3.0 (stable) [12], an open-source Python package for exploring, visualizing, and analyzing human neurophysiological data. MNE-Python was installed as a separate Conda environment (Anaconda Inc., Austin, Texas, USA, 2017) and run using Spyder version 5.4, an open-source integrated development environment for Python. A Python script was used to extract Welch Power Spectral Density (PSD) measurements in the alpha band for all the preprocessed EEG data. Window length was kept at one second (512 data points) with a 50% overlap, as recommended by existing literature. Subsequently, 60 epochs were selected, and the PSD values were averaged, resulting in data being extracted from a cumulative recording duration of two minutes out of the recorded total of around 10 minutes.

Band power asymmetry scores were obtained according to the following definition:

$A = \frac{\mathrm{P}_{left} - \mathrm{P}_{right}}{\mathrm{P}_{left} + \mathrm{P}_{right}} \times 100$,

where A = asymmetry parameter, P_left_ = absolute power in left-sided lead, and P^right^ = absolute power in right-sided lead.

Recording and analysis of short-term HRV indices

For HRV analysis, the lead II ECG recorded simultaneously during EEG recording was used. The signal was recorded at a sampling frequency of 2048 Hz, to which a low-pass filter of 150 Hz was applied. Signal extraction and preprocessing were achieved using Acknowledge 4.2 software (BIOPAC Systems Inc., Goleta, CA, USA). Preprocessing involved the application of a high-pass filter with a threshold of 0.05 Hz and a notch filter centered at 50 Hz. Post filtering, the ECG channel was visually inspected for artifacts and rhythm abnormalities. Then, successive RR intervals were extracted and imported into Kubios HRV Standard version 3.5 (Kubios Oy, Kuopio, Finland) software for HRV analysis. Short-term HRV assessment was performed as per the standards prescribed by the Task Force of the European Society of Cardiology and the North American Society of Pacing and Electrophysiology [13]. Time domain indices, namely, mean R-R interval, mean heart rate (mean HR), SD of all N-N intervals (SDNN), square root of the mean of the sum of the squares of differences between successive N-N intervals (RMSSD), percentage of consecutive NN intervals that differ by more than 50% (pNN₅₀), and frequency domain indices, namely, total power, very low-frequency power (VLF), low-frequency power (LF), high-frequency power (HF), low frequency to high-frequency ratio (LF/HF ratio), LF in normalized units (LF n.u.) and HF in normalized units (HF n.u.) were computed. In HRV analysis, raw R-R intervals are screened, and non-sinus intervals corresponding to arrhythmic or ectopic beats are not included in the analysis, and the resultant set of intervals corresponding to sinus beats are referred to as N-N intervals [13]. The recordings for all participants showed sinus rhythm.

Sample size estimation

From existing literature, a conservative assumption was made regarding Cohen’s f² effect size for the association between the severity of depression, EEG, and HRV parameters to be not less than 0.27 [14]. The sample size was calculated using G*Power version 3.1.9.7 [15] with a fixed model linear multiple regression with R² increase as the statistical test for three potential predictors of the outcome variable, with a level of significance kept at 5% and power at 80%. The estimated sample size was 45 subjects.

Statistical analysis

Data analysis was done using the R statistical language in RStudio version 2023.03.0 [16]. Categorical variables such as gender, education, occupation, and presence of suicidality were expressed as proportions, while continuous variables such as age, HDRS-17 score, HRV parameters, and EEG parameters were expressed as means with SDs or medians with interquartile ranges based on the distribution of data. The Kolmogorov-Smirnov test was used to assess for normality, and Q-Q plots were used to confirm the distribution visually. The inter-group differences in parameters between controls and MDD patients were compared using the independent Student’s t-test or the Mann-Whitney U test, depending on the distribution of the data. The relationship between the severity of depression (HDRS-17 score), EEG alpha asymmetry, and HRV parameters was determined using Pearson’s or Spearman’s rank correlation. Multiple linear regression analysis was used to model the association between HRV parameters, EEG parameters, and HDRS-17 score. All statistical analyses were carried out at a 5% level of significance, where a p-value less than 0.05 was considered statistically significant.

## Results

A total of 45 patients with MDD were included in the test group, and 45 age- and gender-matched healthy individuals were included in the control group. In the MDD group, the youngest patient was 19 years of age and the oldest was 41 years of age. The median age was 28 with an IQR of 12.5. The mean BMI was 23.4 with an SD of 2.5, and the median number of years of education was 14 years with an IQR of six years (Table 1). In the control group, the youngest subject was 22 and the oldest was 38. The control group had a median age of 27 years with an IQR of 10 (Table 1). There was no significant difference in age (Mann-Whitney U test, p-value = 0.749) or BMI (t-test, p-value = 0.504) between the two groups.

**Table 1:** Demographic details of the group of patients with MDD and the age- and gender-matched control group MDD, major depressive disorder

Parameter	Category (if applicable)	Measures in MDD cases (N = 45)	Measures in controls (N = 45)
Age (as median (IQR))		28 (12.5)	27 (10)
Age group (represented as frequency (%) of a total, N = 45)	18-5 years	20 (44.4%)	21 (46.7%)
	26-35 years	14 (31.2%)	16 (35.5%)
	36-45 years	11 (24.4%)	8 (17.8%)
Sex (represented as frequency (%) of a total, N = 45)	Male	28 (62.2%)	28 (62.2%)
	Female	17 (37.8%)	17 (37.8%)
BMI (as mean ± SD)		23.4 ± 2.5	23.8 ± 3.2
Number of years of education (as median (IQR))		14 (6)	21 (2.5)

Among the test group participants, the mean severity of depression assessed with HDRS-17 was 21.78 with an SD of 5.63. On the basis of the severity score, patients were classified into mild (HDRS-17 score ranging from 8 to 16), moderate (HDRS-17 score ranging from 17 to 23), and severe (HDRS-17 score of 24 or greater). Nine subjects (20%, N = 45) had mild depression, 19 subjects (42.2%, N = 45) had moderate depression, and 17 subjects (37.8%, N = 45) had severe depression (Table 2).

**Table 2:** Disease characteristics and medication status for the group of patients with MDD HDRS₁₇, total score as rated with a 17-point Hamilton Depression Rating Scale; MDD, major depressive disorder

Parameter	Category (if applicable)	Measures in patients with MDD
HDRS-17 score (as mean ± SD for a total, N = 45)		21.78 ± 5.63
Duration of illness in weeks (as median (IQR) for a total, N = 45)		4 (5)
Severity of depression (represented as frequency (%) of a total, N = 45)	Mild depression (HDRS₁₇ 8-16)	9 (20%)
	Moderate depression (HDRS₁₇ 17-23)	19 (42.2%)
	Severe depression (HDRS₁₇ >24)	17 (37.8%)
Medication status (represented as frequency (%) of a total, N = 45)	Drug naive	43 (95.6%)
	Drug defaulter	2 (4.4%)

A comparison of HRV indices between the test and control groups revealed significantly low NN₅₀ (p = 0.041) in the test group. Similarly, among the frequency domain indices, LF n.u. and LF/HF ratio were significantly higher, and HF n.u. was significantly lower in the test group compared to the control group (Table 3).

**Table 3:** Comparison of short-term HRV indices between patients with MDD and healthy controls Data are presented as median (IQR). * Considered statistically significant at p < 0.05 HF, high-frequency band (0.15-0.40 Hz) power spectral density; HR, heart rate; HRV, heart rate variability; LF, low-frequency band (0.04-0.15 Hz) power spectral density; LF-HF ratio, the ratio between low-frequency and high-frequency band power spectral densities; MDD, major depressive disorder; n.u., normalized units (relative power); RMSSD, square root of the mean of the sum of the squares of differences between successive NN differences; RR, ECG RR interval; SDNN, SD of NN intervals

HRV parameters	In MDD cases (N = 45) as median (IQR)	In healthy controls (N = 45) as median (IQR)	p-Value
Mean RR interval (ms)	782.17 (192.61)	825.76 (138.04)	0.089
SDNN (ms)	29.99 (35.07)	38.01 (23.18)	0.713
Mean HR (bpm)	76.71 (18.68)	72.66 (12.14)	0.089
RMSSD(ms)	32.47 (42.55)	41.38 (26.69)	0.126
NN₅₀	32 (87.5)	79 (107)	0.041*
pNN₅₀(%)	8.93 (25.2)	22.56 (29.47)	0.072
LF n.u.	58.39 (31.59)	45.13 (14.2)	<0.001*
HF n.u.	41.6 (31.43)	54.63 (14.19)	<0.001*
LF-HF ratio	1.4 (1.72)	0.83 (0.47)	<0.001*

Comparison of EEG alpha power spectral density between MDD patients and healthy controls revealed statistically significantly high alpha PSD in F7 (p = 0.024), T3 (p = 0.007), C3 (p = 0.018), C4 (p = 0.029), T5 (p = 0.033), P4 (p = 0.047), and T6 (p = 0.029) (Table 4).

**Table 4:** Comparison of absolute EEG alpha power spectral density between patients with MDD and healthy controls Data are presented as median (IQR). * Considered statistically significant at p < 0.05 Fp1, Fp2, F3, F4, F7, F8, Cz, C3, C4, Pz, P3, P4, T3, T4, T5, T6, O1, O2, standard electrode position nomenclature as per the international 10-20 system of electrode placement as recommended by the International Federation of Societies of EEG and Clinical Neurophysiology C, central; EEG, electroencephalography; F, frontal; MDD, major depressive disorder; O, occipital; P, parietal; PSD, power spectral density; T, temporal

EEG alpha band PSD parameter	In MDD cases (N = 45) as median (IQR)	In healthy controls (N = 45) as median (IQR)	p-Value
Fp1	2.7 (2.86)	1.77 (2.1)	0.13
Fp2	2.66 (2.67)	1.82 (2.21)	0.159
F7	3.16 (2.97)	2.03 (2.44)	0.024*
F3	1.5 (1.9)	1.18 (1.38)	0.109
Fz	1.21 (1.81)	1.08 (0.98)	0.271
F4	1.42 (1.85)	1.21 (1.42)	0.285
F8	2.59 (2.63)	1.89 (2.19)	0.117
T3	4.16 (5.38)	2.67 (2.79)	0.007*
C3	1.3 (1.87)	0.99 (0.76)	0.018*
Cz	Reference electrode	Reference electrode	-
C4	1.36 (2.48)	0.9 (1.07)	0.029*
T4	3.65 (4.31)	2.48 (3.1)	0.051
T5	7.92 (7.5)	5.04 (4.94)	0.033*
P3	4 (4.75)	2.52 (2.81)	0.081
Pz	2.6 (3.9)	1.54 (2.31)	0.109
P4	4.49 (4.26)	2.52 (3.26)	0.047*
T6	8.03 (8.12)	4.67 (7.84)	0.029*
O1	8.72 (8.23)	6.05 (7.1)	0.124
O2	8.75 (8.45)	5.85 (7.52)	0.126

Alpha asymmetry in the three frontal lead pairs (FP1-FP2, F3-F4, and F7-F8) and T3-T4 was all found to be significantly higher in MDD cases compared to controls (p < 0.001 in frontal, p = 0.002 in T3-T4). No significant differences were found for parietal asymmetry or occipital asymmetry (Table 5).

**Table 5:** Comparison of EEG alpha asymmetry index between patients with MDD and healthy controls ^a^ Data are presented as mean ± SD. ^b^ Data are presented as median (IQR). * Considered statistically significant at p < 0.05 Fp1, Fp2, F3, F4, F7, F8, Cz, C3, C4, Pz, P3, P4, T3, T4, T5, T6, O1, O2, standard electrode position nomenclature as per the international 10-20 system of electrode placement as recommended by the International Federation of Societies of EEG and Clinical Neurophysiology C, central; EEG, electroencephalography; F, frontal; MDD, major depressive disorder; O, occipital; P, parietal; T, temporal

EEG parameters	In MDD cases (N = 45) as mean ± SD or median (IQR)	In healthy controls (N = 45) as mean ± SD or median (IQR)	p-Value
FP1-FP2 asymmetry^a^	2.02 ± 3.62	-0.14 ± 1.81	<0.001*
F3-F4 asymmetry^b^	3.78 (6.51)	-0.09 (2.22)	<0.001*
F7-F8 asymmetry^a^	6.4 ± 6.13	0.56 ± 5.27	<0.001*
C3-C4 asymmetry^b^	-1.69 (16.62)	0.59 (18.16)	0.586
P3-P4 asymmetry^b^	-2.05 (15.55)	-0.21 (10.3)	0.49*
T3-T4 asymmetry^b^	6.17 (11.05)	-0.48 (10.06)	0.002*
T5-T6 asymmetry^a^	-8.57 ± 15.39	-4.79 ± 14.88	0.247*
O1-O2 asymmetry^b^	1.85 (21.52)	-0.02 (11.85)	0.939

Correlation analysis revealed weak to moderate positive correlations between HDRS-17 and age (ρ = 0.313, p = 0.036), duration of illness (ρ = 0.336, p = 0.036), F7-F8 asymmetry (r = 0.364, p = 0.014), LF n.u. (r = 0.349, p = 0.019) and LF/HF ratio (ρ = 0.416, p = 0.005), while a weak negative correlation was observed between HDRS-17 and HF n.u. (r = -0.347, p = 0.019) (Table 6).

**Table 6:** Correlation of severity of depression (HDRS-17 score) with age, education, total duration of illness, EEG alpha asymmetry, and HRV parameters r represents Pearson’s r correlation coefficient. ρ represents Spearman’s rho correlation coefficient. * Considered statistically significant at p < 0.05 Fp1, Fp2, F3, F4, F7, F8, Cz, C3, C4, Pz, P3, P4, T3, T4, T5, T6, O1, O2, standard electrode position nomenclature as per the international 10-20 system of electrode placement as recommended by the International Federation of Societies of EEG and Clinical Neurophysiology C, central; EEG, electroencephalography; F, frontal; HF, high-frequency band (0.15-0.40 Hz) power spectral density; HR, heart rate; HDRS-17, Hamilton Depression Rating Scale 17; HRV, heart rate variability; LF, low-frequency band (0.04-0.15 Hz) power spectral density; LF-HF ratio, the ratio between low-frequency and high-frequency band power spectral densities; MDD, major depressive disorder; n.u., normalized units (relative power); O, occipital; P, parietal; RMSSD, square root of the mean of the sum of the squares of differences between successive NN differences; RR, ECG RR interval; SDNN, SD of NN intervals; T, temporal

Parameter compared to HDRS 17	Correlation coefficient (N = 45)	95% CIs (lower limit, upper limit)	p-Value
Age	ρ = 0.313	(0.029, 0.597)	0.036
Number of years of education	ρ = -0.166	(-0.461, 0.129)	0.276
Illness duration	ρ = 0.336	(0.054, 0.618)	0.024*
FP1-FP2 asymmetry	r = 0.151	(-0.152, 0.456)	0.321
F3-F4 asymmetry	ρ = -0.034	(-0.333, 0.265)	0.824
F7-F8 asymmetry	r = 0.364	(0.095, 0.668)	0.014*
C3-C4 asymmetry	ρ = 0.155	(-0.14, 0.45)	0.265
P3-P4 asymmetry	r = 0.198	(-0.101, 0.502)	0.191
T3-T4 asymmetry	ρ = 0.038	(-0.261, 0.337)	0.802
T5-T6 asymmetry	r = 0.128	(-0.176, 0.434)	0.402
O1-O2 asymmetry	ρ = 0.348	(0.068, 0.628)	0.019*
Mean RR interval	ρ = -0.262	(-0.55, 0.026)	0.082
SDNN	ρ = -0.079	(-0.377, 0.219)	0.606
Mean HR	ρ = 0.262	(0.029, 0.597)	0.082
RMSSD	ρ = -0.147	(-0.443, 0.149)	0.337
NN₅₀	ρ = -0.102	(-0.399, 0.195)	0.506
pNN₅₀	ρ = -0.085	(-0.383, 0.213)	0.579
LF n.u.	r = 0.349	(0.076, 0.653)	0.019*
HF n.u.	r = -0.347	(-0.65, -0.074)	0.019*
LF-HF ratio	ρ = 0.416	(0.144, 0.688)	0.005*

On modeling with multiple linear regression, a model with all six correlates as predictors showed a nonsignificant partial correlation in the case of illness duration, O1-O2 asymmetry, and significant collinearity between LF n.u., HF n.u., and LF/HF-ratio. On further analysis, a model with F7-F8 asymmetry and HF n.u. as predictors was found to be statistically significant while also meeting the assumptions required for multiple linear regression (Durbin-Watson statistic = 1.451, variance inflation factor = 1.018). The multiple linear regression model has an R² of 0.224 (p = 0.005) (Table 7, Table 8). From R², Cohen’s f² effect size can be calculated to be 0.29.

**Table 7:** Multiple linear regression analysis with HDRS-17 score as the outcome variable in relation to F7-F8 alpha asymmetry (EEG) and HF n.u. (HRV) * Considered statistically significant at p < 0.05 EEG, electroencephalography; HDRS-17, Hamilton Depression Rating Scale 17; HF, high-frequency band (0.15-0.40 Hz) power spectral density; HRV, heart rate variability; n.u., normalized units (relative power)

Model	R	R-squared	F-statistic	p-Value
Outcome variable: HDRS-17 score, predictors: F7-F8 alpha asymmetry, HF n.u.	0.473	0.224	6.054	0.005*

**Table 8:** Details of the regression coefficients for the multiple linear regression model with HDRS-17 score as the outcome variable in relation to F7-F8 alpha asymmetry (EEG) and HF n.u. (HRV) * Considered statistically significant at p < 0.05 EEG, electroencephalography; HDRS-17, Hamilton Depression Rating Scale 17; HF, high-frequency band (0.15-0.40 Hz) power spectral density; HRV, heart rate variability; n.u., normalized units (relative power)

Predictor variables	Coefficient (β)	95% CI of β (lower limit, upper limit)	t-value	p-Value
Intercept	23.84	(19.42, 28.25)	10.898	<0.001*
F7-F8 alpha asymmetry	0.298	(0.044, 0.551)	2.364	0.023*
HF n.u.	-0.089	(-0.169, -0.008)	-2.221	0.032*

## Discussion

The pursuit to discover definitive biomarkers for mood disorders has been a particular point of emphasis within psychopathological research. In this study, the utility of EEG and HRV indices as biological markers for the severity of depression in MDD was explored. The primary objective of this research was to elucidate the associations between the severity of MDD, EEG alpha asymmetry, and HRV indices. Weak to moderate correlations were observed between the severity of depression and certain EEG asymmetry, as well as HRV measures. We also found a multiple linear regression model that showed a relationship where the severity of depression could be predicted from HRV HF n.u. and EEG F7-F8 alpha asymmetry.

The control of heart rate and, consequently, HRV is the result of a complex interaction of multiple feedback and regulatory systems, the chief of which is the baroreflex [8]. It is well established that mood and affect can cause variation in heart rate and its variability. A large landmark study by Licht et al. [17] established that depression is associated with significantly lowered HRV. Several other studies have also corroborated the general trend of reduction in HRV in MDD [18-20]. Our findings are aligned with the general consensus regarding HRV in MDD.

We noted an overall trend of reduced HRV in MDD patients compared to healthy controls. With regard to time domain indices, prior studies have shown significantly lower RMSSD, SDNN, and pNN₅₀ in MDD [19]. In our sample, these time domain indices were not significantly different between the MDD and control groups but did show a decreasing trend. However, evidence is present for HRV differences being lower in the drug-naive state, which could explain the lack of significant differences in the time domain indices [21]. Furthermore, the studies showing significant time-domain differences have used longer recordings to determine HRV [21]. Our study used short-term HRV, a single continuous recording of a five-minute duration, and the incongruence could also be due to this difference in methodology. With frequency domain parameters, however, we noted statistically significant differences between MDD and controls. The low-frequency component was higher in MDD, while the high-frequency component was lower, corroborated by a significantly higher LF/HF ratio in MDD. These findings, namely nonsignificant differences in time domain indices with significantly higher LF, LF/HF ratio, and lower HF, align with the results of the study by Jangpani et al. [22], which was also conducted in the South Asian population.

We found significant differences in alpha asymmetry metrics between MDD and controls for the frontal lead pairs (Fp1-Fp2, F3-F4, and F7-F8), with frontal alpha asymmetry (FAA) being positive and higher in the MDD group. Additionally, T3-T4 temporal asymmetry was also found to be positive and significantly higher in MDD. The pattern of FAA that is classically described in the literature is left-sided asymmetry, i.e., increased left-sided alpha power relative to the right, resulting in positive values of FAA [5,23]. For the frontal lead pairs, our findings are consistent with this observation at the group level. The underlying assumption is that MDD is characterized by right prefrontal cortex hyperactivity coupled with left prefrontal cortex hypoactivity [23]. However, there are instances of the opposite pattern being noted, i.e., right-sided frontal asymmetry [23,24]. Six patients with MDD in our data set showed right-sided FAA in the F7-F8 lead pair. Several studies have also found significant parietal asymmetry in MDD compared to controls [14,25].

A weak positive correlation (r = 0.364, p = 0.014) was found between HDRS-17 scores and F7-F8 alpha asymmetry. A recent study by Marcu et al. [25] also noted this relationship in resting state eyes-closed EEG, albeit using MADRS as the scale for scoring the severity of depression. This is to be expected as MADRS and HDRS-17 are highly correlated, showing good convergent validity and allowing for a nearly linear translation between the two [26]. They did not find a correlation when using BDI-II for scoring the severity of depression. Baik et al. [14], who also used BDI-II, did not find correlations between frontal asymmetry and BDI-II. These inconsistencies highlight the need for employing multiple scales simultaneously and performing more rigorous and varied analyses on the underlying electrophysiological data. We discerned a statistically significant multiple linear regression model with the severity of depression, i.e., the HDRS-17 score as the dependent variable, and two predictor variables: HF n.u. and F7-F8 FAA. The coefficients of this model were also statistically significant, implying a meaningful relationship between the severity of depression, HF n.u., and F7-F8 FAA. Nevertheless, the model’s R-squared value was 0.224, suggesting that our model could only account for approximately 22.4% of the variation observed in the HDRS-17 scores. This gives a Cohen’s f² effect size of 0.29, which is considered small [27]. Despite our efforts to construct other models using different HDRS-17 correlates, none were statistically significant, failing at the level of individual model coefficients or the overall model ANOVA. The small effect size is expected since many prior studies on EEG FAA alone have failed to demonstrate a significant relationship with the severity of depression, finding FAA to be relatively stable and independent of clinical condition at the time of recording [2].

In a similar analysis by Baik et al. [14], the researchers split the alpha band into high and low. They noted that HF n.u. and the LF/HF ratio showed a moderating effect only in parietal alpha asymmetry and only in the low alpha band of 8-10 Hz. The moderating effect was not statistically significant for high alpha (11-12 Hz) or total alpha (8-12 Hz) bands. Besides checking for moderation effects, multiple linear regression modeling was also attempted but was statistically insignificant. In our sample, the severity of depression was not correlated to parietal alpha asymmetry scores. However, in contrast to their study, all parameters in our study were recorded in a drug-free state. Since antidepressant medications have been shown to affect HRV [6] and EEG alpha asymmetry [28], it could be one possible source of variation that could explain the inconsistent results controlling for which may have resulted in the statistically significant linear regression model in our study.

In this study, the alpha band was analyzed as the total alpha power and was not split for the analysis. Also, our sample had a lower age bandwidth, i.e., 19-41 years compared to 20-65 years in the study by Baik et al. [14], and had more male subjects with a male:female ratio of 1.64 compared to 0.38 in the study by Baik et al. [14], which could have contributed to further variation in the results [4]. Methodological and analysis pipeline differences are known to influence quantitative EEG measures [29]. The differences in the tools used to quantify the severity of depression can also lead to differences in outcomes [25,30].

Strengths

Patients were drug naive or had a minimum of six weeks of drug-free interval at the time of recording, controlling for the effects that pharmacotherapy can have on EEG and HRV indices. Additionally, the assessment and recording of parameters were carried out prior to the start of treatment (in 43 out of 45 subjects). Thus, the assessment of the severity of depression, which is subjective to the psychological state of the patients, would not be confounded by improvement due to treatment.

Limitations

The study had the following limitations. The study sample displayed a nonnormal distribution of age, with a skew toward younger participants. Therefore, our findings may be more representative of and directly applicable to younger patients with MDD. Only a single recording of five minutes of ECG (short term) was used for HRV analysis, which has been shown to have higher situational variation. We could not implement a mechanism to objectively record respiratory rate during HRV assessment due to resource constraints. Furthermore, BMI and cardiovascular training were not controlled for. Also, we only used a single measurement scale for the assessment of severity, used convenience sampling, and did not control for the potential influence of socioeconomic and lifestyle-related confounders, all of which limit the context of our interpretations. The study is also from a single center with participants belonging to the southern part of India; hence, generalizability to other population groups is uncertain. We also do not have follow-up data, due to which the stability of these findings cannot be commented upon.

Implications and future directions

The effect size of our predictive model is small (Cohen’s f²=0.29) and can only predict 22.4% of the variation in the measured severity of depression. Thus, the model on its own is of limited clinical predictive utility. However, this is evidence of the presence of predictive patterns for the severity of depression within EEG and HRV measurements, especially in the drug-naive state. Since the effect sizes are small and the number of possible contributing variables is large, they may not be conducive to manual elucidation, making it a prime candidate for the use of machine learning. Future research should explore the application of machine learning algorithms to develop predictive models for MDD. Additionally, we only used HDRS for assessment of severity; whether such patterns exist across other scales of measurement needs exploration. There is also the scope for further studies with more granular means of classifying the severity of depression as well as investigations into any relationships between specific symptoms of MDD and EEG or HRV parameters.

## Conclusions

Our findings provide evidence of a relationship between the severity of depression and two objective physiological measures, namely HRV HF n.u. and EEG FAA, in the form of a multiple linear regression model. The combination of HRV HF n.u. and EEG F7-F8 FAA could explain 22.4% of the variation in severity of depression, as quantified by HDRS-17, mainly in newly diagnosed drug-naive patients of MDD at presentation.

We also found significant differences between depressed and euthymic controls matched for age and gender in frequency domain parameters of HRV as well as F7-F8 FAA. This represents another step in the pursuit of elucidating the complex array of factors that contribute to the heterogeneity of the EEG and HRV profile of MDD and its severity. However, the difficulty of delineating and elucidating these factors is evident, given the small effect size of our model, possibly implying other sources of variation moderating the parameters involved and limiting its direct clinical utility. However, there is scope for exploration into using multiple rating scales, more granular measurement of symptoms, and the use of machine learning algorithms to develop models with greater predictive capability.
